# Emergency Department Use by Women Experiencing Homelessness in Los Angeles, California, USA

**DOI:** 10.1089/whr.2021.0142

**Published:** 2022-06-13

**Authors:** Christine Samuel-Nakamura, Mary-Lynn Brecht, Rachel Arbing

**Affiliations:** School of Nursing, University of California, Los Angeles (UCLA), Los Angeles, CA, USA.

**Keywords:** homelessness, women's health, Emergency Department use, disability, traumatic brain injury

## Abstract

**Background:**

This article reports on the use of hospital Emergency Departments (EDs) in women experiencing homelessness in Los Angeles, California. Women 18 years of age or older were recruited from homeless day centers in Los Angeles to participate in this study.

**Materials and Methods:**

A self-report questionnaire on health status, demographics, and emergency service use was completed by study participants.

**Results:**

In this study of women experiencing homelessness, 64% utilized the ED within the past year. The mean number of ED use was 3.63 (range 0–20) visits in the past year. Higher frequency visits were significantly associated with several mental health conditions (*p* = 0.016), physical disability (*p* = 0.001), and traumatic brain injury (*p* = 0.013).

**Conclusions:**

The physical and psychological impacts of the homelessness experience can be enormous, affecting the homeless individually and collectively. Study findings may help to understand how to improve services that support and meet the needs of women experiencing homelessness such as patient and family-centered care and trauma-informed care in the ED.

## Introduction

Frequent use of the hospital Emergency Department (ED) is common among those experiencing homelessness.^1,2^ Since women experiencing homelessness are considered a high-risk group and have more complex health problems, and prolonged hospitalization rates that contribute significantly to health care costs, their unique perspective, and health care experiences were sought for this study. Broadly, using the homelessness definition set by the United States Department of Housing and Urban Development,^3^ homelessness in the United States has reached upward of 575,000 unhoused people in 1 year. In the city and county of Los Angeles (LA), homelessness is as high as 50,000–60,000 on any given night, of which 31% are women.

The number of people experiencing homelessness over the age of 62 years grew by 20% in 2020, and grew even further to 67% for those over age 55 years.^4^ Many disparities of social determinants exist that contribute to homelessness such as poverty, unemployment,^5^ social exclusion, and marginalization.^6^ This population experiences more chronic health conditions, disability, and elevated morbidity and mortality than the general population.^5,7^

Multiple studies have demonstrated that homeless individuals are prone to frequent use of ED services, often reporting inadequate health care and follow-up.^8,9^ Disease burden has a direct effect on health care use.^8^ For the current study, characterizing morbidities and comorbidities among the homeless that use ED services regularly may offer insights into strategies to manage their long-term care and follow-up more effectively. Furthermore, study findings may help to understand how to improve services that support and meet the needs of women experiencing homelessness who often have more comorbidities than counterpart men.^10^

There exists a knowledge gap in the ED usage characteristics of women experiencing homelessness, in particular, a subset of underrepresented groups such as American Indian/Alaskan Natives (AI/AN) and those of Asian descent. This was a descriptive cross-sectional study that sought to test the hypothesis that various health conditions reported by women experiencing homelessness have an association to ED frequency of use.

## Materials and Methods

### Study population

Adult women experiencing homelessness 18 years of age and older were recruited *via* handout study flyers in the downtown LA area. Flyers were posted at homeless clinics, organizations, and at public establishments near homeless shelters. A site to administer the survey at a local day center for women was established. Potential participants were screened for study eligibility (women, age 18+ years, and experiencing homelessness in LA County). Recruitment was ongoing over a 12-month period from 2019 to 2020.

Eligible women (*N* = 47) were verbally told about the study and were consented in writing, told they did not have to participate or answer any question(s) they did not want to, and that services would not be withheld if they chose not to participate. Participants were provided a written consent form to sign if they agreed to participate. Study incentives provided to participants were self-care items such as toothbrush/paste, socks, backpack, soap, and shampoo. Personal identifiers were not collected on the surveys. The University of California, Los Angeles (UCLA) Institutional Review Board provided approval to undertake the study.

### Questionnaire/survey instrument

This descriptive, cross-sectional study of ED use by women experiencing homelessness employed a self-administered 30–45-minute survey. The study survey was written in English at the sixth grade Flesch–Kinkaid Grade Level. The demographic information and questions regarding health diagnoses were similar to that collected by the Los Angeles Homeless Services Authority (LAHSA).^11^ It was coordinated during the designated lunch hour at a daytime shelter in a private room, the survey collected data on demographics, history of physical and mental health problems, and ED use. The self-report measures included:

- Physical and mental health diagnoses: health care provider diagnosis of problem/illness, type of health or mental health problem, substance abuse and violence experiences.
- Health care frequency of use: number of times the ED or hospital was visited during the past 12 months. For the purposes of this study, frequent use of the ED was defined as more than four visits per year.^12,13^

Each category listed above was composed primarily of items with multiple choice responses, however, the ED frequency of use question was an open-ended question. To maintain confidentiality, participants were not asked the name or the location of the ED/hospital they visited, or if the ED visits were for the same health-related problem.

### Statistical analysis

Data were analyzed using Statistical Package for the Social Science (IBM SPSS Statistics for Windows, Version 26.0; SPSS).^14^ For the variable “race,” categories are not mutually exclusive, so they were treated as separate binary variables. Different types of employment status are shown in Table 1.

**Table 1.** Distribution of Emergency Department Use by Demographic Indices for Homeless Women in Los Angeles, California, USA (*n* = 42)

Demographic indices	Reported ED use (%), *n* = 30	Reported no ED use (%), *n* = 12
Educational attainment		
<HS	6 (20)	0 (0)
HS graduate/GED	7 (23)	6 (50)
Some college	12 (40)	3 (25)
4-year college	4 (13)	1 (8)
No answer/declined	1 (<1)	2 (17)
Employment status		
Retired	2 (7)	1 (8)
Unemployed, student	1 (3)	0 (0)
Unemployed, not looking	2 (7)	1 (8)
Unemployed, seeking employment	7 (23)	3 (25)
Part-time/temporary/seasonal	1 (3)	1 (8)
Disability+part-time/temporary/seasonal	1 (3)	0 (0)
Disability only (no work, not retired)	8 (27)	3 (25)
No answer/“None”/”Declined”	8 (27)	3 (25)
Race/ethnicity^a^		
Hispanic^*^	14 (47)	1 (8)
White	11 (37)	1 (8)
Black	16 (53)	6 (50)
American Indian/Alaska Native	5 (17)	0 (0)
Asian	3 (10)	0 (0)
Other	5 (17)	2 (17)

Missing data assessment: significant difference between those who did not answer the question, “did you use ED services?” (*n* = 5) and those who answered “no” or “yes” to the question (*n* = 42) found for education: 80% of those with missing ED answer also gave “no” answer for education, whereas only 7% of nonmissing ED answer group gave “no” answer for education. There were no differences in other demographics.

^a^ Race/ethnicity categories are not mutually exclusive. Percentages indicate those answering “yes” to the specific category; respondents could answer “yes” to more than one category.

^*^ *p* < 0.05 comparing ED use to no ED use.

ED, Emergency Department; GED, General Educational Development; HS, high school.

Women reporting ED visits were compared with those reporting no visits on demographic characteristics with *p*-values calculated from chi-square tests of independence. A *p*-value of <0.05 was considered statistically significant and demonstrates evidence of association with ED use with demographics. The Mann–Whitney *U* test with exact *p*-values compared the frequency of ED visits (using ranks instead of the actual number) between women with diagnosis versus women without diagnosis. Only those (*n* = 30) who provided a specific number of ED visits or reported no ED use (*n* = 12; counted as zero visits) were included in this analysis. Effect sizes were calculated from Mann–Whitney test statistics and translated to Cohen's *d*.^15^ In addition to exact *p*-values for Mann–Whitney *U* (listed in Table 2), we also computed *p*-values adjusted for the multiple tests in this analysis using a modified Benjamini–Hochberg correction to maintain a false discovery rate of 0.05^16^; all unadjusted *p*-values <0.05 were also significant with adjusted *p* < 0.05 (not shown in Table 2).

**Table 2.** Associations Between Emergency Department Visits and Reported Medical Diagnoses

Reported medical diagnoses	z* statistic for Mann–Whitney *U	*p*	Effect size d	Number of ED visits (*n* = 30)							
				Those using the ED with reported medical diagnoses				Those using the ED without the reported medical diagnoses			
				*n*	Mean ± SD	Median (IQR)	Min–Max	*n*	Mean ± SD	Median (IQR)	Min–Max
Mental health disorders	2.458	0.016	0.96	11	7.2 ± 7.8	3 (14)	0–20	19	1.6 ± 2.8	0 (2)	0–12
Developmental disability	1.338	0.225	0.48	2	11.0 ± 12.7	11 (18)	2–20	28	3.1 ± 5.0	1.5 (3)	0–20
TBI	2.477	0.011	0.97	3	14.3 ± 9.8	20 (17)	3–20	27	2.4 ± 3.9	1 (3)	0–15
Physical disability	3.276	<0.001	1.41	12	6.4 ± 6.3	3 (8.5)	0–20	18	1.8 ± 4.7	0 (2)	0–20
TBI or physical disability	3.847	<0.001	1.84	13	7.5 ± 7.1	3 (9)	0–20	17	0.7 ± 1.0	0 (2)	0–3
Problem with drug and/or alcohol use	0.127	0.924	0.05	7	2.7 ± 3.7	1 (5)	0–10	23	3.9 ± 6.3	2 (3)	0–20
Dating violence or stalking	1.400	0.187	0.51	15	4.9 ± 6.3	2 (10)	0–20	15	2.3 ± 5.1	0 (3)	0–20
Physical and sexual abuse by a partner/spouse	2.343	0.022	0.90	7	6.6 ± 6.9	3 (10)	2–20	23	2.7 ± 5.2	0 (3)	0–20
Physical and sexual abuse by parent, guardian, or relative	0.993	0.360	0.36	7	6.7 ± 9.7	2 (20)	0–20	23	2.7 ± 4.1	1 (3)	0–20

IQR, interquartile range; TBI, traumatic brain injury; SD, standard deviation.

A comparison of those included in ED frequency analysis and those not included because of missing data showed no significant differences in demographics or health/abuse conditions. Because there was a small sample size for several specific mental health disorder diagnoses (such as depression, bipolar disorder, post-traumatic stress disorder, schizophrenia, and any form of depression), serious mental health problems were combined into one broad category (“mental health conditions”) for analysis. Specific types of abuse were also combined: physical and/or sexual abuse by intimate partners, physical/sexual abuse by relatives, and “dating violence” (physical violence and stalking).

## Results

### Study population characteristics

Forty-seven women experiencing homelessness 18 years of age and older participated in the study. Five of these participants did not respond to the question on ED frequency of use and were not included in these results. Slightly more than one-half of the women self-reported being single (*n* = 23, not shown in Table 1). The sample was racially/ethnically diverse, self-identifying as Black (*n* = 22), Hispanic (*n* = 15), White (*n* = 12), AI/AN (*n* = 5), of Asian descent (*n* = 3), or “Other” (*n* = 7). Fifty-three percent of those reporting ED visits reported “some college” or possessing a 4-year college degree, 30% reported they were disabled, and 23% were “seeking employment” (Table 1). Women with ED visits did not differ significantly from those with no ED visits on demographic characteristics, except for those that self-identified as Hispanic.

### Reported medical diagnoses

Thirty women (71%) reported utilizing the ED at least once during the last year. Among those reporting specific frequency of ED use or reporting no use, the number of visits ranged from 0 to 20 with a mean of 3.63 (SD = 5.77) visits and a median of 2 (interquartile range = 3) in the past 12 months. For the subsample (*n* = 18) who reported at least one ED visit, the mean number of visits was 6.06 (SD = 6.42, range 1–20).

A statistically significant difference (and large effect size) in frequency of ED visits was found for women with several reported health conditions compared with those without these conditions. These conditions included mental health disorders (*p* = 0.016, *d* = 0.96), physical disability (*p* < 0.001, *d* = 1.41), and traumatic brain injury (TBI; *p* = 0.011, *d* = 0.97) (Table 2). The relationship of frequency of use of ED services to reported problems with substance use (drug and alcohol use; *p* = 0.924) was not found to be statistically significant.

Those women who reported having a history of any physical and/or sexual abuse (*p* = 0.022, *d* = 0.90) by a spouse or intimate partner reported greater number of ED visits than counterpart women; this includes those experiencing dating violence and/or stalking (*p* = 0.187, *d* = 0.51; Table 2). Other reported types of abuse by parents, guardians, or relatives (neglect, physical and sexual abuse) were not found to be significantly (*p* = 0.36) related to number of ED visits, and a small effect was found (*d* = 0.36). An association between violence and abuse experienced by women and ED use has implications for placement following homelessness status, as a safe environment is an essential element of trauma-informed care as well as multifaceted follow-up care.

## Discussion

ED services play a vital role in the delivery of medical care to patients in times of need. When the ED becomes the only source of medical care, due to lack of access to primary care, overuse of the ED becomes costly and burdensome. Examining the characteristics of women experiencing homelessness who frequent the ED provides information on their physical and mental health, high-risk behaviors, as well as the social and environmental context of their daily lives living on the streets or in temporary shelters.

Homelessness affects many major U.S. cities across the nation. Los Angeles is home to the second largest population of people who are experiencing homelessness, second only to New York City.^3^ In LA, studies of groups experiencing homelessness have reported the racial distribution to be primarily Black^16^ or Hispanic, followed by Whites.^17,18^ Similarly, those women who use the ED frequently in this study had a analogous racial distribution. The distribution of those experiencing homelessness and barriers to health care can vary greatly between minority ethnic groups and reveal cultural inequities.^18,19^

AI/AN and those of Asian descent subgroups remain unrepresented, underreported, and uncharacterized and need to be brought to the forefront of similar research on homelessness. For instance, a large U.S. and Canadian study reported more than half of Indigenous mothers and caretakers reported having experienced homelessness in their lifetimes and those that were homeless manifested more physical, mental health, and substance abuse problems.^20^ Similarly, Wille et al.^21^ found that a myriad of unique homeless health care barriers can exist for AI/AN populations. Similar racial inequalities and cultural differences were noted elsewhere in Latina women.^18,19^

Multiple ED visits by women experiencing homelessness were significantly associated with mental health conditions, physical disability, and TBI. Moore et al.^7^ reported several risk factors for homelessness; they include mental illness,^22,23^ chronic illness, cognitive impairment, and violence. Although Nilsson et al.^23^ found no association between TBI and risk of homelessness, Stubbs et al.^24^ reported the prevalence of TBI to be 53.1% (95% CI 46.4–59.7; *I*^2^ = 97%) among those experiencing homeless and marginally housed persons and demonstrated an association between TBI and worsened mental and physical health, higher risk of suicide and suicidality, increased health care access, memory issues, and increased involvement of the criminal justice system. Binder et al.^25^ concluded that TBI can both be a cause and consequence of homelessness.

It is beyond the scope of this article to discuss the types and extent of TBI for the current study population, however, the impact of TBI on health and function are extensive and further examination is warranted in this high-risk population.

Adverse life events in childhood and adulthood consisting of physical abuse were found by other researchers to be risk factors for becoming homeless as well as having psychiatric problems.^17,22^ Studies have reported that domestic violence and sexual abuse affect more women experiencing homelessness than men, and their primary reasons for becoming homeless were found to be family and economic-related causes.^26^ This study demonstrated an association between violence and abuse experiences among women experiencing homelessness and ED use, particularly in those reporting abuse by a spouse or partner. Study participants reporting abuse by intimate partners and spouses showed more frequent ED use.

Physical disability was found to be significantly associated with ED frequency of use (*p* = 0.001). There was a moderate subset of ED users who reported their disability under occupational status (*n* = 9). There are only a few recent studies that have examined “physical disability” in groups experiencing homelessness. Several studies examined various types of disability such as “psychiatric,” “intellectual disability,”^7^ and “sensory disability.”^27^ For the current study, disability was categorized as two primary types: “physical disability” or “developmental disability.” Certainly, some of the listed diagnoses (*i.e.*, TBI, mental health disorders) in the current study may overlap and fall into various types of disabilities whether they are “physical” or otherwise. For instance, TBI can fall into the category of physical disability but also overlap with neuropsychological or psychological disorders^28^—depending on the extent and nature of the head injury, patient characteristics, or comorbidities or additional injuries.^29^

In the current study, when TBI was analyzed alone, statistical significance was found; furthermore, when TBI was combined with physical disability, it was found to be statistically significant at a *p* level of <0.001 (*d* = 1.84, Table 2). One study found an association between physical disability, multiple health problems, and frequent use of ED services in those with a history of abuse.^30^ Studies did conclude that people with disabilities have a higher risk of becoming homeless than the general population.^27^ Physical disability was one of several key vulnerabilities of homelessness reported in one study on U.S. Veterans.^31^ It is important to define the type of disability in studies for adequate characterization, measurement, and reporting.

The study findings support the importance of utilizing trauma-informed care,^32^ a practice that emphasizes a culture of safety, empowerment and healing. Providing adequate recognition and screening for trauma, providing patient-centered and controlled care are essential.^33^ Espousing collaborative care that fosters empowerment and resilience are key elements in the trauma-informed care model.

Patient-centered care with service integration has also been successful across the country^34^ where more than 250 “HealthCare for the Homeless” (funded by the Health Resources and Service Administration) sites feature multidisciplinary teams to address the myriad of health care and social needs using a plethora of dedicated care strategies. These programs developed partnerships with major health care centers and with other key sectors such as housing, criminal justice, and social services. Emphasizing the importance of primary care services in conjunction with other key resources and promotion of continuity of care upon discharge from the ED and aftercare are essential to address the increase frequency of use in this high-risk population.

The negative impacts of COVID-19 have been great upon those experiencing homelessness. The COVID-19 pandemic hit LA during the current study period in March of 2020. Social distancing, quarantining, and frequent handwashing is prohibitive in these population settings due to a general lack of resources, and other social and medical inequalities.^35^ According to a capacity needs study undertaken early in the pandemic,^36^ estimates demonstrated that homeless individuals with COVID-19 were anticipated to have an increased 4.3% (or 21,295 person) need for hospitalization, a 0.6%–4.2% increase need for critical care and an anticipated increased fatality of 0.3%–1.9% in the homeless population of LA county and New York City.

A study by Riley et al.^37^ reported that women experiencing homelessness did not decrease their ED use during the pandemic (dissimilar to the general population) and those that had challenges in accessing drug-use treatment were eight times more likely to use the ED. The recommendations by the authors included providing access to housing and low-barrier health and substance use treatment services during “big event” public health crises. The full and long-term impacts of COVID-19 in those experiencing homelessness has yet to be fully determined and reported.

There are U.S. federal mandates to provide emergency care despite patients' inability to pay. Ku et al.^12^ found that ED users were most frequently “discharged to street” and the rest were admitted for hospitalization or left without being treated. In January of 2019, the Homeless Discharge Senate Bill (SB 1152) was enacted in California to promote adequate provisions for discharge planning from outpatient and inpatient settings for homeless individuals.^38^ Inappropriate discharge or “dumping” of individuals experiencing homelessness with inadequate resources were the impetus for SB 1152. The provisions were intended to provide those experiencing homelessness to return to the community with adequate resources, shelter, treatment, and other supportive services.

Initially, identification of homeless individuals, their postdischarge destination, providing transportation, adequate clothing, screening for health coverage, and infectious disease referral and vaccination were required; as of July 2019, expanded requirements include communication with postdischarge entities, a patient discharge log and method of compliance documentation. This study was initiated and completed after the implementation of SB 1152, and whether it had an influence on the current study results are unknown. Similar work examining the short and long-term impacts of SB 1152 on ED accessibility/discharge and its impacts are needed.

Our homelessness study characterized ED visits by women in a large, U.S. urban geographic area during a 1-year period and found a high frequency of use compared with similar studies.^12,13^ Female study participants were primarily single, mostly Black, Hispanic, or White reporting a high frequency of disability. Significant differences were found by comparing those women with and without diagnosis in terms of frequency of ED use; these differences were found in mental health conditions, physical disability, TBI, and reported history of physical and sexual abuse by a spouse or intimate partner.

## Limitations

This study had several limitations. Data were collected from a small sample size from a single-site location and would have been strengthened by a comprehensive and larger sample size; the onset of the COVID-19 pandemic halted the current study enrollment, which may have inhibited some individuals in participating in the study. Self-report was an issue particularly for reporting the number of ED visits. Whiteside et al.^39^ found 45% of respondents inaccurately reported the number of visits to ED in a 1-year period. Feasible solutions to consider for future studies were identified by similar studies.^40,41^ For instance, utilizing simplified questions that ascertain ED accessibility in the past 6 months rather than focusing on reports of open-ended number of visits may have improved data accuracy. The majority (>75%) of the study sample possessed more than a high school and General Educational Development-level education, however, offering the use of language translation (Spanish and other languages) may have been beneficial.

## Conclusions

The physical and psychological impacts of the homeless experience can be enormous, affecting the homeless individually and collectively. The majority of women experiencing homelessness who participated in the study reported that they frequently used the ED and those using ED services reported a mean of 3.63 times in the last 12 months; the ED frequency of use is comparable to those defined by Ku et al.^12^ and LaCalle et al.^13^ (>4 ED visits/year). The high incidences of repeat ED use and frequent hospital use indicates the need for ongoing changes to health care resources and follow-up aftercare.

The association between ED use with health problems and life-long abuse brought about by parents or guardians, and in some cases in later years by intimate partners or spouses, was shown to be statistically significant. Underserved populations experiencing homelessness, particularly women, are at risk of experiencing violence, psychosocial problems, and worsened health care outcomes. Efforts to address the physical and mental health needs of those experiencing homelessness require a better understanding of the environment of women, including health care accessibility and resource availability.

A subset of underrepresented women experiencing homelessness was identified that would benefit from further analysis to improve access to resources that are more culturally appropriate, inclusive, and equitable. There is a need to adequately train health care providers, patients, and their families on the effects of homelessness and strategies for improved health outcomes in this population.

## Abbreviations Used

95% CI: 95% confidence interval
AI/AN: American Indian/Alaskan Natives
ED: Emergency Department
GED: General Educational Development
HS: high school
IQR: interquartile range
LA: Los Angeles
SD: standard deviation
TBI: traumatic brain injury
UCLA: University of California, Los Angeles
